# Changes in oral health indicators due to implementation of the National Health Insurance Services coverage for first molar dental sealant for children and adolescents in South Korea

**DOI:** 10.1186/s12903-020-01201-8

**Published:** 2020-07-29

**Authors:** Jin-Sun Choi, Deuk-Sang Ma

**Affiliations:** Department of Preventive and Public Health Dentistry, College of Dentistry & Research Institute of Oral Science, Gangneung-Wonju National University, 7 Jukheon-gil, Gangneung City, Gangwon Province, 25457, South Korea

**Keywords:** Dental sealant, Health insurance coverage, Oral health indicators

## Abstract

**Background:**

In South Korea, dental sealant was included in the National Health Insurance Services (NHIS) coverage for the first molar for ages 6–14 in December 2009. The second molar was included in 2012, and the age of insurance coverage was extended to under 18 in 2013. This study aimed to verify the effectiveness of an NHIS dental sealant coverage policy for children and adolescents by comparing the changes in first molar oral health indicators before and after policy implementation.

**Methods:**

The Korea National Health and Nutrition Examination Survey data were analyzed; the fourth period (2007–2009) provided data for before and the sixth period (2013–2015) provided data for after policy implementation. The proportion of individuals with first-molar sealant, decay-missing-filled first molar permanent teeth, and single crowns in the group aged 11–20 years were calculated. Data were analyzed using chi-square for complex samples and the complex samples general linear model. In addition, complex-sample logistic regression analysis was performed to confirm the association between factors.

**Results:** Compared with non-beneficiaries, among policy beneficiaries, sealant ownership increased by 7.7% (from 27.8 to 35.5, *P* < 0.001), and the number of permanent teeth with sealant per capita increased by approximately 0.4 to 0.8 (*P* < 0.001). The proportion of individuals with decay-missing-filled permanent teeth decreased by 9.1% (from 68.4 to 59.3, *P* < 0.001), and the average decay-missing-filled permanent teeth index per person decreased by approximately 2.0 to 1.5 (*P* < 0.001). The rate of single-crown holders decreased by 2.7% (from 8.7 to 6.0, *P* > 0.05), and the average single-crown index decreased by approximately 0.11 to 0.08 per person(*P* > 0.05). The number of sealants increased with age and household income (*P* < 0.001). The mother’s education level affected sealant experience (*P* < 0.05). The caries rate was higher in females and older respondents (*P* < 0.001).

**Conclusions:**

The sealant covered by NHIS contributed to decreasing dental caries in Korea. However, policies that can reduce oral health inequality should also be considered, and a follow-up study is required for long-term sealant maintenance in Korea.

## Background

Dental caries is the most common oral disease in childhood [1] and can lead to tooth loss if left untreated [2]. Moreover, oral care in this period is particularly important because it can influence oral health in adulthood. In 1981, the oral health objective of the World Health Organization (WHO) and World Dental Federation (FDI) was to decrease the average DMFT index of 12-year-olds to below 3 by 2000. Since then, the increasing efforts related to oral health in countries worldwide enabled 68% of 184 countries to reduce their decay-missing-filled teeth (DMFT) index to below three in 2000. However, the remaining countries continue to have DMFT index values higher than three [3]. Furthermore, according to the Global Burden of Disease Study 2017, it is estimated that about 2.3 billion people worldwide suffer from caries of permanent teeth, while 530 million suffer from caries of the deciduous teeth. In the decade between 2007 and 2017, the number of years of living with disability is estimated to have increased by 9.4% (from 8.6 to 10.3) and 4.9% (from 3.1 to 6.3) for caries of the permanent and deciduous teeth, respectively. Therefore, tooth decay is an important oral health problem experienced by numerous people globally [4].

To reduce the prevalence of dental caries among children and adolescents in South Korea (hereafter Korea), the government implemented a free-sealant oral health program through public health centers from 2002 to 2010 [5]. These publicly based oral health programs played an important role in reducing the DMFT index for permanent teeth among 12-year-olds from 3.25 in 2003 to 2.17 in 2006 [6].

Korea implements the National Health Insurance Services (NHIS) in the country through the National Health Insurance Corporation (NHIC). All Koreans pay health insurance premiums based on their income and receive the same benefits. The Ministry of Health and Welfare regularly updates the treatments and standards necessary for supporting NHIC. Therefore, the treatments are divided into those paid by insurance and those that are not. If adopted by the NHIS, part of the treatment cost is paid by the government, and the remaining is paid by the patient (out-of-pocket payment) [7].

Among the dental treatments available in Korea, health insurance covers radiation diagnosis, endodontic treatment, extraction and other oral surgery, periodontal treatment (include scaling), temporomandibular joint treatment, dentures and implants for the elderly, and resin filling for children [7].

As the preventive effectiveness of dental sealant has been verified, this treatment was included in the NHIS for children aged 6 to 14 from December 2009 onward [8]. In October 2012, the second molar was additionally covered, and in July 2013, the age of coverage was extended to under 18 years [8]. At the start of the health insurance, the out-of-pocket payment ranged from 30 to 60%, while that by the government (insurance benefit) ranged from 40 to 70% [8].

Previous studies [9, 10] have shown that sealant treatment is increasing with the expansion of sealant health insurance. However, it has been confirmed that differences in the use of sealant treatment according to socioeconomic factors still exist [9, 10]. This may be attributed to the fact that government-led sealant projects are rapidly curtailed or abolished after reimbursement [11], resulting in a decrease in the supply of sealant to vulnerable groups.

On the other hand, the permanent caries prevalence rate in 12-year-olds decreased slightly by 0.9% over 3 years, but the decrease observed from 7.8% in 2015 (about 5 years after sealant reimbursement) [12] to 6.9% in 2018 (about 8 years after the reimbursement) [13] was not significant.

Given these phenomena, it is necessary to evaluate the effect of sealant treatment after the implementation of the reimbursement policy for children and adolescents on the change in their oral health indicators. Therefore, this study aimed to identify the effectiveness of the sealant reimbursement policy for children and adolescents by comparing the differences in first molar oral health indicators, as well as differences according to sociodemographic characteristics.

## Methods

### Study participants

In this study, we obtained approval to analyze the raw data from the Korea National Health and Nutrition Examination Survey (KNHANES), which can be accessed through its website [14]. The KNHANES is a national survey that identifies the current state and related trends of the health and nutritional status of the population, establishes target indicators and evaluation data for the health care plan for Koreans, and calculates suitable health indicators for comparison with other countries [14]. To improve the representativeness of the sample and the accuracy of the estimations, the sample area was extracted using the multi-stage stratified colony probability extraction method, which is a complex-sample design method [15].

### Study variables and measurement

Examinations for the KNHANES are conducted at the Mobile Examination Center and include physical measurements, blood pressure and pulse measurements, blood and urine tests, oral examinations, pulmonary function tests, eye tests, and grip tests [14]. The oral examination is performed by dentists who have completed the calibration for disease control [16]. The calibration verifies the reliability of dentists by measuring their kappa values. Dentists’ mean kappa values averaged 0.714–0.938 in the fourth (2007–2009) KNHANES period and 0.876–0.958 in the sixth (2013–2015) period. We used the results of oral examinations performed according to WHO standards by dentists in a Mobile Examination Center for the analysis [17–22].

The study was approved by the Committee on Research Ethics at the National Statistical Office and the Centers for Disease Control and Management (Approval number: 2007-02CON-04-P, 2008-04EXP-01-C, 2009-01CON-03-2C, 2013-07CON-03-4C, 2013-12EXP-03-5C) [23].

For the data analysis in the present study, the fourth period (2007–2009) of the KNHANES was selected as the dataset before the sealant reimbursement policy, and the sixth period (2013–2015) as the dataset after the reimbursement policy was implemented. Participants’ age was selected to be 11–20 years in order to compare oral health indicators after 5–6 years after policy implementation in 2010, based on an age of 6–14 of those who received reimbursement benefits.

Thus, we categorized those without NHIS benefits as “non-beneficiaries” (the fourth period, 2007–2009) and those who receive NHIS benefits as “beneficiaries” (the sixth period, 2013–2015).

The target tooth was selected as the first molar, which is covered by the sealant reimbursement policy. An indicator was created by converting the code of the tooth condition in the oral examination section of the KNHANES examination. The dependent variables were first molar with or without sealant, permanent caries, and single crown. Independent variables included gender (male, female), age (11–15, 16–20), region (urban, rural), household income (I: lower, II: lower-middle, III: middle-upper, and IV: upper), father’s education level (less than high school, more than college), and mother’s education level (less than high school, more than college).

### Statistical analysis

Statistical analysis was conducted using SPSS 23.0 (SPSS Inc., Chicago, IL, USA) and STATA 13.0 (Copyright Stata Corp LP, USA) statistical package. The proportion of individuals with first-molar sealant, decay-missing-filled permanent teeth, and single-crown among individuals aged 11–20 years were calculated, and a complex-sample chi-square test was conducted to identify significant differences according to the factors under investigation. In addition, the average number of instances of sealant, decay-missing-filled permanent teeth, and single-crown for the first molar in participants aged 11–20 years were calculated. In order to compare the difference between “non-beneficiaries” and “beneficiaries” regarding sealant treatment, and to compare and analyze the differences according to the related factors, a descriptive statistical analysis considering complex samples for each independent variable was conducted using the complex samples general linear model. The variables with statistical significance were presented using a complex samples logistic regression model to confirm the association between variables and to calculate the odds ratios (ORs) and 95% confidence levels (CI).

The definitions of the terms are as follows:

1. Sealant rate (%): the percentage of dental sealant holders in first molars
2. Sealant index: the number of dental sealant permanent per capita in first molars
3. DMF rate (%): the percentage of decay-missing-filled permanent teeth in first molars
4. DMFT index: the average decay-missing-filled permanent teeth index per person in first molars
5. Crown rate (%): the percentage of single-crown holders in first molars
6. Crown index: single-crown index per person in first molars

## Results

Table 1 shows the characteristics of the participants, including the number of non-beneficiaries and beneficiaries based on gender, age, region, household income, and father and mother’s education levels.

**Table 1:** Characteristics of Subjects

Classification		Non-beneficiaries (2007–2009 year)		Beneficiaries (2013–2015 year)	
		N	Wt(%)^a^	N	Wt(%)^a^
Gender	Male	1574	51.9	1333	51.8
	Female	1518	48.1	1258	48.2
Age	11–15 years	1902	53.9	1420	46.6
	16–20 years	1190	46.1	1171	53.4
Region	Urban	2516	83.8	2201	85.2
	Rural	576	16.2	390	14.8
Household income	I(lower)	386	13.7	327	13.6
	II (Lower-middle)	711	23.6	669	27.4
	III (Middle-upper)	956	31.8	817	30.9
	IV (Upper)	958	30.9	753	28.1
Father’s education level	Less than high school	200	71.7	192	57.9
	More than college	90	28.3	145	42.1
Mother’s education level	Less than high school	245	87.1	227	67.1
	More than college	44	12.9	115	32.9

^a^Weighted value

Table 2 shows the application of sealant reimbursement among Korean children and adolescents, and the differences in sealant treatment according to sociodemographic characteristics.

**Table 2:** The application of dental sealant reimbursement among Korean children and adolescents, and the differences in sealant treatment according to sociodemographic characteristics

Classification		Sealant rate^a^ (%)				Sealant index^b^ (Mean ± SE^d^)			
		Non-beneficiaries		Beneficiaries		Non-beneficiaries		Beneficiaries	
Total		27.8		35.5	***c	0.45 ± 0.05		0.83 ± 0.03	***c
Gender	Male	26.7	NS	35.1	NS	0.62 ± 0.03	NS	0.87 ± 0.05	NS
	Female	29.1		35.9		0.58 ± 0.04		0.78 ± 0.05	
Age	11–15 years	34.9	***	43.3	***	0.82 ± 0.04	***	1.13 ± 0.05	***
	16–20 years	19.5		28.3		0.35 ± 0.03		0.55 ± 0.05	
Region	Urban	27.9	NS	35.5	NS	0.60 ± 0.03	NS	0.84 ± 0.04	NS
	Rural	27.6		35.5		0.61 ± 0.08		0.76 ± 0.09	
Household income	I(lower)	17.6	***	26.5	***	0.35 ± 0.05	***	0.60 ± 0.09	*
	II (Lower-middle)	22.4		32.1		0.50 ± 0.06		0.78 ± 0.07	
	III (Middle-upper)	27.7		36.3		0.57 ± 0.04		0.80 ± 0.06	
	IV (Upper)	37.0		42.2		0.83 ± 0.05		1.00 ± 0.07	
Father’s education level	Less than high school	14.3	NS	18.8	NS	0.20 ± 0.05	NS	0.29 ± 0.06	NS
	More than college	24.7		23.2		0.39 ± 0.10		0.48 ± 0.11	
Mother’s education level	Less than high school	18.2	NS	15.9	*	0.26 ± 0.05	NS	0.33 ± 0.06	NS
	More than college	12.8		30.5		0.24 ± 0.13		0.53 ± 0.11	

^a^The data were analyzed by Complex samples chi-square test

^b^The data were analyzed by Complex samples general linear model

^c^The difference between Non-beneficiaries and Beneficiaries (by Complex samples chi-square test and general linear model)

^d^Standard error

^***^p < 0.001,^**^p < 0.01,^*^p < 0.05,^NS^*p > 0.05*

Compared with the non-beneficiaries, the sealant rate among the beneficiaries of the reimbursement policy increased by 7.7% (27.8 to 35.5%). The number of sealant permanent per capita doubled, 0.45 in non-beneficiaries and 0.83 in beneficiaries, and the difference between the two groups was significant (*P* < 0.001). Sealant retention (rate and index) was higher in women, younger age groups, for those whose father had higher education, and those with higher household income. Age and household income were significant factors (*P* < 0.001). Mothers’ education levels were higher in the lower level of education among non-beneficiaries, and were not statistically significant. For the beneficiaries, the higher the mother’s education level, the higher the sealant retention; this association was found to be significant (*P* < 0.05).

Table 3 shows the application of sealant reimbursement among Korean children and adolescents, and the differences in decay-missing-filled permanent teeth index according to sociodemographic characteristics.

**Table 3:** The application of dental sealant reimbursement among Korean children and adolescents, and the differences in decay-missing-filled permanent teeth retention according to sociodemographic characteristics

Classification		DMF rate^a^ (%)				DMFT index^b^ (Mean ± SE^d^)			
		Non-beneficiaries		Beneficiaries		Non-beneficiaries		Beneficiaries	
Total		68.4		59.3	*** c	2.09 ± 0.10		1.57 ± 0.04	*** c
Gender	Male	62.2	***	54.9	***	1.74 ± 0.05	***	1.38 ± 0.05	***
	Female	75.1		64.0		2.19 ± 0.05		1.77 ± 0.06	
Age	11–15 years	60.3	***	50.1	***	1.64 ± 0.04	***	1.26 ± 0.05	***
	16–20 years	77.9		67.5		2.32 ± 0.05		1.84 ± 0.06	
Region	Urban	68.5	NS	59.6	NS	1.97 ± 0.04	NS	1.57 ± 0.05	NS
	Rural	67.6		57.3		1.88 ± 0.08		1.53 ± 0.11	
Household income	I(lower)	68.4	NS	62.3	NS	2.03 ± 0.10	NS	1.70 ±0.12	*
	II (Lower-middle)	69.7		60.3		2.01 ± 0.07		1.63 ±.0.09	
	III (Middle-upper)	69.2		61.4		1.98 ± 0.07		1.63 ±0.07	
	IV (Upper)	66.8		54.8		1.85 ± 0.06		1.38 ±0.07	
Father’s education level	Less than high school	81.9	NS	73.3	NS	2.47 ± 0.11	NS	1.95 ±0.12	NS
	More than college	79.8		68.6		2.28 ± 0.17		1.93 ±0.15	
Mother’s education level	Less than high school	81.6	NS	72.0	NS	2.39 ± 0.10	NS	1.95 ±0.12	NS
	More than college	83.4		71.3		2.70 ± 0.26		1.98 ±0.16	

^a^The data were analyzed by Complex samples chi-square test

^b^The data were analyzed by Complex samples general linear model

^c^ The difference between Non-beneficiaries and Beneficiaries (by Complex samples chi-square test and general linear model)

^d^Standard error

^***^p < 0.001,^**^p < 0.01,^*^p < 0.05,^NS^*p > 0.05*

Compared with the non-beneficiaries, the DMFT rate among the beneficiaries decreased by 9.1% (68.4% vs. 59.3%). The DMFT index per person was 2.09 for the non-beneficiaries and 1.57 for the beneficiaries; this indicates a decrease of about a quarter, which was significant (*P* < 0.001). The decay-missing-filled permanent teeth retention (DMF rate, DMFT index) was higher in women (*P* < 0.001), in the older age group, those living in the urban area, and those whose father’s education was lower. In the non-beneficiaries, the association was significant for gender and age group (*P* < 0.001), and in the beneficiaries, for Household income (*P* < 0.05).

Table 4 shows the application of sealant reimbursement among Korean children and adolescents, and the differences in single-crown treatment according to sociodemographic characteristics.

**Table 4:** The application of dental sealant reimbursement among Korean children and adolescents, and the differences in Single-crown permanent teeth retention according to sociodemographic characteristics

Classification		Crown rate^a^ (%)				Crown index^b^ (Mean ± SE^d^)			
		Non-beneficiaries		Beneficiaries		Non-beneficiaries		Beneficiaries	
Total		8.7		6.0	NS c	0.11 ± 0.02		0.08 ± 0.00	NS c
Gender	Male	7.5	NS	5.7	NS	0.10 ± 0.01	*	0.07 ± 0.01	NS
	Female	9.9		6.3		0.14 ± 0.01		0.09 ± 0.01	
Age Group	11–15 years	4.5	***	3.2	***	0.06 ± 0.00	***	0.04 ± 0.00	***
	16–20 years	13.6		8.5		0.19 ± 0.01		0.12 ± 0.01	
Region	Urban	9.0	NS	5.8	NS	0.12 ± 0.01	NS	0.08 ± 0.01	NS
	Rural	7.1		7.0		0.10 ± 0.02		0.10 ± 0.02	
Household income	I(lower)	7.0	NS	6.4	NS	0.11 ± 0.02	NS	0.08 ± 0.02	*
	II (Lower-middle)	11.1		6.7		0.17 ± 0.02		0.10 ± 0.02	
	III (Middle-upper)	8.2		6.5		0.10 ± 0.01		0.09 ± 0.01	
	IV (Upper)	8.5		4.9		0.11 ± 0.01		0.05 ± 0.01	
Father’s education level	Less than high school	11.5	NS	11.8	NS	0.18 ± 0.04	NS	0.17 ± 0.04	NS
	More than college	15.0		16.7		0.18 ± 0.57		0.21 ± 0.05	
Mother’s education level	Less than high school	12.4	NS	14.6	NS	0.20 ± 0.03	NS	0.21 ± 0.04	NS
	More than college	9.3		16.6		0.09 ± 0.06		0.21 ± 0.05	

^a^The data were analyzed by Complex samples chi-square test

^b^The data were analyzed by Complex samples general linear model

^c^ The difference between Non-beneficiaries and Beneficiaries (by Complex samples chi-square test and general linear model)

^d^Standard error

^***^p < 0.001,^**^p < 0.01,^*^p < 0.05,^NS^*p > 0.05*

Compared with the non-beneficiaries, the Crown rate fell by 2.7% (8.7% vs 6.0%). The Crown index per person decreased by 0.03, with 0.11 for the non-beneficiaries and 0.08 for the beneficiaries, but the difference was not significant. Single-crown holdings were higher in females (*P* < 0.05), those in the higher age group(*P* < 0.001), and those with a higher level of father’s education (*P* > 0.05).

Table 5 shows the results of the analysis of the association using a Complex samples logistic regression model between the sealant rate, household income, and mother’s education level.

**Table 5:** Odds ratios of sealant rate among the groups categorized by household income and mother’s education level (95% CI)

Classification	Non-beneficiaries		Beneficiaries	
	Model 1 ^a^	Model 2 ^b^	Model 1 ^a^	Model 2 ^b^
**Sealant**				
Household income				
I(lower)	0.379 (0.264–0.544) ^***^	0. 982 (0.318–3.029)	0.552 (0.373–0.818) ^**^	0.845 (0.287–2.487)
II (Lower-middle)	0.493 (0.364–0.666) ^***^	0.604 (0.230–1.582)	0.671 (0.503–0.896) ^**^	0.729 (0.324–1.640)
III (Middle-upper)	0.648 (0.495–0.847) ^**^	0.913 (0.379–2.199)	0.751 (0.570–0.991) ^*^	0.626 (0.274–1.427)
IV (Upper)	Ref. 1.000	Ref. 1.000	Ref. 1.000	Ref. 1.000
Mother’s education level				
Less than high school	1.579 (0.583–4.275)	3.115 (1.060–9.150) ^*^	0.439 (0.226–0.851)^**^	0.393 (0.177–0.871) ^*^
More than college	Ref. 1.000	Ref. 1.000	Ref. 1.000	Ref. 1.000

The data were analyzed by Complex Samples Logistic Regression

^a^Adjusted for age

^b^Adjusted for gender, age, region, father’s education level, mother’s education level, household income

^***^p < 0.001,^**^p < 0.01,^*^p < 0.05,^NS^*p > 0.05*

In Model 1, for both non-beneficiaries (ORs = I:0.37, II:0.49, III:0.64, *P* < 0.001, *P* < 0.01) and beneficiaries (ORs = I:0.55, II:0.67, III:0.75, *P* < 0.01, *P* < 0.05), the lower the household income, the lower the sealant rate, with the statistical significance maintained. In Model 2, the gap between the odds ratios of the beneficiaries (ORs = I:0.84, II:0.72, III:0.62, *P* > 0.05) decreased more compared to the non-beneficiaries (ORs = I:0.98, II:0.60, III:0.91, *P* > 0.05); however, inequality still existed without statistical significance (*P* > 0.05). Further, in Model 2, ORs of the sealant experience was higher for non-beneficiaries when the mother’s education level was low (ORs = 3.11, *P* < 0.05); however, it was higher for beneficiaries when the mother’s education level was high (ORs = 0.39, *P* < 0.05).

## Discussion

Sealant reimbursement for the first molar began in 2009 for individuals aged 6–14 in December 2009. Through two revisions, the second molar was added to the coverage in October 2012. In July 2013, the age range was expanded to those under 18 years of age. We aimed to identify the effectiveness of the sealant reimbursement policy for the first molar for those aged 11–20 years (who benefited from the beginning of reimbursement following the implementation of the sealant coverage in Korea) by identifying differences in oral health indices between beneficiaries and non-beneficiaries as well as associations with sociodemographic characteristics.

First, the sealant holder rate increased by 7.7% (27.8% for the non-beneficiaries and 35.5% for the beneficiaries) and the mean number of first molars with sealant per capita doubled (from 0.45 for non-beneficiaries to 0.83 for beneficiaries). This increase is estimated to be affected by the sealant reimbursement policy. However, this is low compared with the two-thirds of Danish 15-year-olds who have at least one sealant [24], as Korea has not yet reached one sealant per person. The main reason for the increase in the sealant holding rate not meeting the expectations was the low-income groups, as there was a relatively high cost-sharing ratio of 30–60% for the first 7 years (from December 2009 to September 2017), which may have raised the threshold for dental access. Before reimbursement, the cost of one sealant is approximately USD 34, of which 30–60% is approximately $10–20. It can be estimated that the cost will be about $40 to $80 per person, provided that all four first molars are treated per person. These costs are considered to be very expensive for the vulnerable groups. Prior studies have shown that high partial copayments will be particularly burdensome for low-income individuals [25]. The relationship between socioeconomic factors and sealant has weakened after the reimbursement policy, but still existed [9].

In this study, we found a significant difference in sealant retention according to the mother’s education level and household income. Similar past studies have reported that higher household income is associated with a higher sealant retention rate [26]. For beneficiaries, the mother’s education level demonstrated statistical significance in both Models 1 and 2, in which the covariates were adjusted. Therefore, mother’s education level can be considered an important variable in South Korea. Previous studies have shown that the prevention of oral disease is greatly influenced by the mother [27], and that the mother’s knowledge and attitude are related to the child’s carious and oral hygiene [28]. Additionally, a previous study showed that the higher the parents’ education level, the higher the sealant experience [29].

Meanwhile, in the case of non-beneficiaries, the sealant rate was high for mothers with lower education levels. Before the implementation of the sealant NHIS, the government had, since 2002, been providing sealant for free to about 200,000 elementary school students in rural areas; later, the scope was expanded to include elementary school students from vulnerable groups in cities. This contributed significantly to the elimination of inequality in the use of sealant. However, after the inclusion of sealant in NHIS, the budget for the free sealant program was cut completely [30].

After the inclusion of dental sealant in health insurance in Korea, as parents have the right to decide the treatment for their children, they have to visit the dental institution to obtain the sealant and pay for part of the treatment. In a previous study, the mother’s socioeconomic status and oral-health screening were found to be related to her child’s experience related to sealant [31]. This indicates that a mother’s knowledge of and interest in oral health will also affect her child’s oral health. Therefore, in addition to the provision of sealant by NHIS, there is a need for oral health policies focusing on school health centers where sealant is supplied to children and adolescents who have difficulty accessing dentistry.

Meanwhile, some developed countries provide free preventive dental services for children and adolescents. In the French dental system, children and adults are eligible for free preventive dental services every 3 years from 3 to 24 years of age [32]. In addition, Sweden regards children’s dental care as part of the nation’s universal welfare, and services for dental health for children and adolescents up to 19 years of age are provided free of charge [33].

In 2012, Korea piloted the family dentist system for vulnerable children under 18 years of age living in Seoul for the first time [34]. Since then, this system has been introduced and implemented in some areas. The family dentist system in Korea is a scheme that provides dental medical services such as oral examination, preventive care, and treatment in connection with the public health center and local dental clinic for low-income children [35]. This system has shown positive effects in terms of oral health awareness and behavior, and it is also very positive that students who have low access to dental healthcare and who do not receive dental services can be beneficiaries of the system [36]. Since 2020, the family dentist system for children has been piloted as a government-led project. In addition, since October 2017, deductibles have been reduced from 30 to 10% because of sealant reimbursement [37]. Nevertheless, in order to provide dental preventive services to vulnerable groups who do not benefit from the reimbursement system, schemes such as the family dentist system for children should be promoted, and measures to supplement the limitations and minimize problems between the systems should be implemented.

The proportion of individuals with decay-missing-filled permanent teeth decreased by about quarter-fold in the beneficiaries compared to the non-beneficiaries—2.09 in the non-beneficiaries and 1.57 in the beneficiaries. In addition, the single-crown retention rate decreased 2.7% (8.7% for the non-beneficiaries and 6.0% for the beneficiaries). A study conducted in Finland also found that sealants in dental public health policy reduced the occurrence of dental caries [38].

The number of single crowns per person decreased by 0.03 (from 0.11 to 0.08), but this was not statistically significant. In other words, both the occurrence of caries and fixed dentures decreased, but these were not significant changes when compared to values before the reimbursement policy. In addition, according to the outpatient ranking of multi-frequency diseases in Korea, dental caries in 2010–2018 ranked 6-7th, which indicates no significant change over 8 years [39]. It is supposed that dental caries is decreasing in children and adolescents, but the caries that have advanced to adulthood and need treatment have not been reduced.

As a result, there seems to be a need to determine how long sealant treatment in children and adolescents can last into adulthood. Some previous studies have shown that the retention and lifespan of sealants act as a beneficial factor in the prevention of tooth decay [40, 41]. That is, after sealant treatment, if left out partially, the risk of caries seems inevitable. Choi et al. insisted on the factors that can improve retention of sealant treatment and asserted the importance of follow-up management to institutionalize and manage return visits after treatment [42]. Although sealant is less costly and more effective as a preventive policy than the expensive post-treatment of caries, the caries prevention effect of sealant will not last into adulthood without considering the loss and maintenance of sealant. In the future, a follow-up system that can ensure sealant retention will need to be implemented.

The DMF rate, DMFT index, and Cr index is higher in women than in men. This finding is the same as that of Jung et al., who reported that female students were at higher risk of experiencing tooth decay than male students [43]. This is because women are exposed to the risk of dental caries more than men as they are more likely to develop permanent teeth, including the first molars, earlier than men [44]. Furthermore, girls are more likely than boys to consume sugary snacks [45]. Therefore, it is necessary to perform a follow-up study to verify the oral health conditions and influencing factors by gender.

In all three indicators, there was no significant difference between cities and rural and urban areas. In 2002, the Korean government implemented an oral health program for providing free sealant to about 200,000 elementary school students in rural areas through health centers [30]. It is considered that this rural-oriented oral health program contributed to reducing the regional differences. However, Choi et al. reported that the supply of sealants differed among regions according to dental access [25]. Therefore, it is necessary to study the changes in oral health indicators in various regions by considering variables related to dental access, such as the extent of the region, and the numbers of dental institutions and workers.

NHIS policies have had a positive effect on public health in Korea [46]. However, addressing health inequality in the country requires additional efforts to reduce medical costs for the vulnerable [47].

The present study used KNHANES data for calculating dental health index statistics required by international organizations (OECD, WHO, etc.) and comparing them between countries, and analyzed dental disease patterns that are representative of Koreans. However, the study has some limitations. First, KNHANES is confined to cross-sectional studies and not cohort studies. Therefore, although the difference between oral health indicators and demographic characteristics was identified, causality could not be determined. Furthermore, the second molar added during the course of NHIS was not included in the present study’s analysis. This may have resulted in underestimating the supply of dental sealant. In the future, we should study the causal relationship between dental sealant experience and socioeconomic factors, including the second molar. In addition, since the out-of-pocket payment for dental sealant has been reduced from 30 to 10%, continuous monitoring of the dental sealant experience will be required.

## Conclusions

Sealant treatment for the first molar in children and adolescents aged 11–20 years was significantly higher in reimbursement policy beneficiaries compared to non-beneficiaries, and caries was significantly lower. In addition, differences in sealant and caries indicators according to sociodemographic characteristics were also identified. Therefore, a system to prevent and treat dental caries based on universal health coverage should be established, and a follow-up system is needed to monitor the retention of sealant.

## Data Availability

The datasets generated and/or analysed during the current study are available in the [Korea National Health and Nutrition Examination Survey] repository, [https://knhanes.cdc.go.kr/knhanes/sub03/sub03_01.do].

## Abbreviations

NHIS: National Health Insurance Servies
NHIC: National Health Insurance Corporation
KNHNES: Korea National Health and Nutrition Examination Survey
